# Effect of Clinicopathological Characteristics on the Outcomes of Topical 5-Aminolevulinic Acid Photodynamic Therapy in Patients with Cervical High-Grade Squamous Intraepithelial Lesions (HSIL/CIN2): A Retrospective Cohort Study

**DOI:** 10.3390/biomedicines12102255

**Published:** 2024-10-03

**Authors:** Yingting Wei, Jing Niu, Liying Gu, Zubei Hong, Zhouzhou Bao, Lihua Qiu

**Affiliations:** 1 Department of Obstetrics and Gynecology, Ren Ji Hospital, School of Medicine, Shanghai Jiao Tong University, Shanghai 200127, China; tinawyting@126.com (Y.W.);; 2 Shanghai Key Laboratory of Gynecologic Oncology, Ren Ji Hospital, School of Medicine, Shanghai Jiao Tong University, Shanghai 200127, China; 3 State Key Laboratory of Oncogenes and Related Genes, Shanghai Cancer Institute, Ren Ji Hospital, School of Medicine, Shanghai Jiao Tong University, Shanghai 200127, China

**Keywords:** cervical high-grade squamous intraepithelial lesions, cervical intraepithelial neoplasia, 5-aminolevulinic acid, photodynamic therapy, high-risk HPV

## Abstract

Background: Minimally-invasive 5-aminolevulinic acid photodynamic therapy (ALA-PDT) is used for treating cervical high-grade squamous intraepithelial lesions (HSIL/CIN2). The purpose of this study was to analyze the factors affecting the efficacy of ALA-PDT in the treatment of cervical HSIL/CIN2 in order to guide physicians in making appropriate treatment decisions. Methods: A retrospective study including 69 female patients with pathologically diagnosed HSIL/CIN2 was conducted. Patients were given six doses of 20% ALA-PDT at 7–14-day intervals. Cytology, HPV testing, colposcopy, and pathology were performed before treatment and at 6-month follow-up after treatment to assess efficacy. The main outcome of this study was the regression of HSIL/CIN2 and the clearance of high-risk HPV (hrHPV) infection after ALA-PDT treatment. Clinicopathological characteristics were collected to analyze the factors affecting the effectiveness of ALA-PDT treatment for HSIL/CIN2. Results: Between the successful and failed lesion regression group, there was a significant difference in sleeping disorders (*p* < 0.05). Between the successful and failed hrHPV clearance group, no statistically significant factors were found. With sensitivity values of 0.556 and 0.778, respectively, multivariate analysis showed that current smoking and sleeping disorders were independent prognostics of failure in lesion regression after ALA-PDT treatment. Conclusions: Smoking and sleep disorders were independent risk factors for failure in HSIL/CIN2 regression following ALA-PDT, suggesting the need for careful consideration of ALA-PDT for patients with these conditions.

## 1. Introduction

Cervical cancer ranks as the fourth most common malignancy among women, with approximately 600,000 new cases and 340,000 related deaths occurring globally each year [1]. The persistent infection with high-risk human papillomavirus (hrHPV) is strongly associated with the development of cervical cancer. While most hrHPV infections are transient and can be cleared by the immune system, persistent infections can lead to cervical intraepithelial neoplasia (CIN) and ultimately invasive cancer. CIN is classified based on the depth of cervical epithelial involvement into CIN1, CIN2, and CIN3, with CIN2 and CIN3 considered high-grade squamous intraepithelial lesions (HSIL). Each grade of CIN exhibits distinct biological behaviors, and the risk of progression varies. At least 12% of CIN3 cases will advance to invasive cancer, while 18% of CIN2 cases progress to CIN3 and invasive cancer [2,3]. Notably, 11% of untreated CIN2 patients under the age of 30 progress within two years [4]. Thus, timely intervention for patients with cervical HSIL is critical to prevent the onset of cervical cancer. The preferred treatment for cervical HSIL is excisional therapy, such as cold-knife conization or loop electrosurgical excision (LEEP). However, these procedures can lead to postoperative complications, including cervical stenosis, adhesions, cervical endometriosis, and cervical insufficiency, which may increase the risk of adverse pregnancy outcomes [5]. Therefore, for women with cervical HSIL who wish to preserve their fertility, a non-invasive therapeutic intervention that reduces the progression rate of CIN2 to CIN3+ is highly desirable.

In recent years, photodynamic therapy (PDT) has been gradually applied in the treatment of a variety of benign and malignant diseases [6,7]. PDT mediated by 5-aminolevulinic acid (5-ALA) is a minimally invasive treatment for CIN. Different from ablation or excision, ALA-PDT uses photodynamic responses to selectively destroy lesions for therapeutic purposes. Local application of ALA-PDT has the advantages of less trauma, less adverse reactions, good selectivity, repeatability, and rapid recovery. The histological remission rate of HSIL/CIN2 treated with ALA-PDT ranges from 77.8% to 91% [8]. Despite these promising results, some patients with HSIL do not benefit from ALA-PDT and may even progress to invasive cancer. Administering conservative treatment without thorough consideration can exacerbate their condition and miss the optimal treatment window.

Therefore, analyzing the factors influencing ALA-PDT outcomes in cervical HSIL patients is essential to identify the most effective treatment strategies. In this study, we collected data on 13 clinicopathological characteristics and investigated their potential predictive ability for outcomes in cervical HSIL patients following ALA-PDT.

## 2. Materials and Methods

### 2.1. Study Design and Study Population

This was a retrospective study. From March 2019 to October 2023, women with hrHPV infection, a pathologic diagnosis of cervical HSIL/CIN2, complete clinical data, and who had received ALA-PDT at the Gynecology Department of Renji Hospital, affiliated with Shanghai Jiao Tong University School of Medicine, were selected. All participants completed a questionnaire administered by a specialist physician. The questionnaire covered demographics such as age, smoking and drinking habits, HPV vaccination status, sleep disorders, number of births and miscarriages, as well as details regarding HPV infection type, cervical cytology results, pathology findings, and laboratory tests such as platelet–lymphocyte ratio (PLR) and neutrophil–lymphocyte ratio (NLR). Those with incomplete questionnaire information and follow-up results were excluded.

### 2.2. HPV Testing and Cytological Test

HPV genotyping was performed through the flow-through hybridization-based 21 HPV GenoArray Diagnostic Kit (Hybribio Ltd., Chaozhou, China) to identify and stratify HPV infection into the 15 high-risk types (HPV 16, 18, 31, 33, 35, 39, 45, 51, 52, 53, 56, 58, 59, 66, and 68) and six low-risk types (6, 11, 42, 43, 44, and 81). We classified HPV infections into the following two categories: HPV16/18 infections and non-HPV16/18 infections. Additionally, we differentiated between multiple-type HPV infections and single-type HPV infections. Cytological tests were performed using liquid-based cytology (ThinPrep cytological test, TCT). The results were categorized as negative for intraepithelial lesion or malignancy (NILM), atypical squamous cells of undetermined significance (ASCUS), low-grade squamous intraepithelial lesion (LSIL), HSIL, atypical squamous cells that could not exclude HSIL, and cervical cancer.

### 2.3. Photodynamic Therapy

To prepare the 20% ALA mixture, 354 mg of 5-ALA powder (Fudan Zhangjiang Bio-Pharm Co., Ltd., Shanghai, China) was combined with 1.5 mL of thermogel (stored at 4 °C). Sterile cotton pieces soaked in the ALA mixture were applied to the cervix and incubated for four hours in the dark. Subsequently, light irradiation at 635 nm and a dose of 100 J/cm^2^ (LD600-C; Wuhan Yage Photo-Electronic Co., Ltd., Wuhan, China) was applied to the cervical surface and the endocervical canal for 30 min respectively. This treatment regimen was repeated six times at intervals of 7–14 days.

### 2.4. Follow-Up and Effect Evaluation

The primary outcomes measured were the regression of HSIL/CIN2 lesions and the clearance of hrHPV infection at the 6-month follow-up after ALA-PDT treatment. The specific grouping criteria were as follows:

1. Lesion: The Success Group was defined as regression of HSIL/CIN2, indicated by either a negative pathological diagnosis or a reduction in histological severity compared to the initial state after ALA-PDT treatment. The Failed Group was defined as persistence of HSIL/CIN2 (no change in histological diagnosis) or progression to CIN3+ at follow-up.
2. hrHPV: The Success Group was defined as conversion from baseline hrHPV-positive to hrHPV-negative at follow-up. The Failed Group was defined as hrHPV remaining positive at follow-up compared to the baseline.

### 2.5. Statistical Analysis

The collected data were analyzed using IBM SPSS software (Version 26.0, SPSS Inc., Chicago, IL, USA). Chi-square, Fisher’s exact, and t tests were conducted to evaluate the difference in the incidence of different outcome groups, respectively. Univariate and multivariate analyses were utilized to find independent predictive factors related to the outcome of HSIL/CIN2 patients undergoing ALA-PDT.

### 2.6. Ethical Considerations

All procedures adhered to the ethical standards of the Institutional and National Research Committee as well as the 1964 Declaration of Helsinki and its subsequent amendments or comparable ethical standards. Informed consent was obtained from all participants included in the study. The study received approval from the Ethics Committee of Renji Hospital, affiliated with Shanghai Jiao Tong University School of Medicine (Committee’s reference number KY2021-248-B).

## 3. Results

We enrolled 69 eligible patients with cervical HSIL/CIN2 who received ALA-PDT into this study and gathered 13 clinicopathological characteristics for analysis.

### 3.1. Analysis of Factors Influencing the Effect of ALA-PDT on HSIL/CIN2 Regression

Among the 69 patients, 60 had experienced histologic regression following ALA-PDT at the 6-month follow-up.

As shown in Table 1, the regression of HSIL/CIN2 was significantly more challenging in patients with sleep disorders compared to those without (65.0% vs. 95.9%, *p* = 0.002). Additionally, patients who were current smokers appeared to have a lower lesion regression rate compared to non-smokers (70.6% vs. 92.3%, *p* = 0.058). There was no significant difference between the two groups in terms of age, drinking history, HPV vaccination, history of childbirth or abortion, PLR, NLR, multiple HPV type infections, HPV16/18 infection, and TCT results (*p* > 0.05). Additionally, ALA-PDT combined with CO_2_ lasers did not show a significantly different influence on the outcome compared to ALA-PDT alone (*p* = 0.357).

### 3.2. Analysis of Factors Influencing the Effect of ALA-PDT on hrHPV Clearance

Among the 69 patients, 44 achieved successful clearance of hrHPV, while 25 had persistent hrHPV infection at the 6-month follow-up. As shown in Table 2, current smoking was associated with a lower hrHPV clearance rate, but this difference was not statistically significant (*p* = 0.099). Additionally, results showed no statistically significant differences between the two groups for the other 12 clinicopathological characteristics.

### 3.3. Univariate and Multivariate Regression Analyses of Factors Influencing the Efficacy of ALA-PDT

We further conducted univariate and multivariate regression analyses to identify independent prognostic factors for the efficacy of ALA-PDT in treating HSIL/CIN2. We employed a stepwise multiple linear regression model to analyze variables with a significance level of *p* < 0.05 from the univariate analysis. As shown in Table 3, first, univariate regression analysis was conducted, revealing associations between current smoking, sleep disorders, and lesion efficacy (*p* < 0.05). Subsequent multivariate regression analysis further indicated that both factors are independent risk factors for poorer histological prognosis (*p* = 0.046 and *p* = 0.039, respectively). Current smoking was strongly associated with a lack of lesion regression, with sensitivity and specificity of 0.556 and 0.800, respectively. Similarly, patients with sleep disorders showed a higher risk of a lack of lesion regression compared to those without sleep disorders, with sensitivity and specificity of 0.778 and 0.783, respectively. For the hrHPV clearance, the univariate analysis showed no statistically significant clinicopathological characteristics (Table 4).

## 4. Discussion

The application of HPV vaccines, regular screening, and timely treatment of cervical precancerous lesions are effective preventive measures for cervical cancer. Untreated HSIL/CIN2 patients have a probability of developing cervical cancer [4]. Studies indicate that the majority of patients with cervical HSIL benefit from ALA-PDT, although a small number may not respond effectively [8,9]. Therefore, evaluating factors influencing ALA-PDT efficacy in cervical HSIL patients can aid in selecting more appropriate treatments. In this study, 13 clinicopathological variables were analyzed.

Smoking has been extensively studied for its association with various diseases. Reports have shown that smoking can cause DNA strand breaks, promote viral amplification in cervical cells infected with hrHPV, and enhance the expression of HPV E6 and E7 proteins [10,11]. A meta-analysis on Japanese women demonstrated that smoking was strongly associated with cervical cancer [10]. Reports also indicate that smoking increases the risk of recurrence of genital warts and HSIL [11]. Building on this, our analysis identified current smoking as an independent risk factor for failure in lesion regression following ALA-PDT. However, current smoking did not significantly affect hrHPV clearance in HSIL/CIN2 patients after ALA-PDT. Further studies with larger sample sizes and longer follow-up periods are warranted to validate these findings.

Numerous studies have demonstrated a connection between sleep and HPV infection. Short sleep durations may increase HPV infection likelihood [12]. Enhancement of sleep quality during HPV infection is assumed to feedback to the immune system to promote host defense [13]. In particular, slow-wave sleep can support immune system recovery [14]. Further understanding of the casual relationship between sleep deprivation and immune deregulation would help to identify individuals at risk of disease and to prevent adverse health outcomes [15]. However, another study found that the quality of sleep had no bearing on HPV infection [16]. The results of this study indicated that sleep significantly influenced lesion regression following ALA-PDT but did not affect hrHPV clearance. Sleep disorders were identified as an independent risk factor for poorer lesion outcomes in HSIL/CIN2 patients treated with ALA-PDT. These findings underscore that HSIL/CIN2 patients with better sleep quality may derive greater benefit from ALA-PDT.

Our previous studies proved that age was a risk factor for ALA-PDT, with the >50 age group showing a higher lesion progression rate and lower HPV remission rate than the <50 age group [17]. However, age did not affect the outcomes of ALA-PDT in patients with HSIL/CIN2 in this study. This may be attributed to the fact that the majority of patients were younger than 50 years old (66/69, 95.65%), reflecting the preference of young women with fertility concerns opting for ALA-PDT. 

Drinking habits and HPV vaccination are known clinical factors that influence HPV infection [18,19,20,21]. A history of childbirth and abortion is also believed to impact vaginal health. We were interested in how these factors might affect the efficacy of ALA-PDT in HSIL/CIN2 patients. Our results showed no significant difference between the groups that achieved success and those that did not in terms of drinking habits, HPV vaccination status, or history of childbirth or abortion. However, further analysis with larger or multi-center sample sizes is needed to derive conclusive findings. Additionally, stratified analysis based on specific childbirth or abortion histories and types of HPV vaccines warrants further research.

HPV testing and the TCT are common laboratory indicators for cervical lesions. A previous study demonstrated that HPV 16/18 positive cases can benefit most from ALA-PDT, and the HPV clearance rate in patients with multiple-type HPV infection was significantly lower than that in patients with single-type HPV infection [22]. The TCT has some degree of cervical lesion presentation and CIN/HSIL diagnostic value; however, in this study, HPV16/18 infection, multiple-type HPV infections, and TCT results were not found to be associated with the outcome of ALA-PDT treatment for HSIL/CIN2, which may be due to the variations in methods or patient populations. The NLR and PLR of blood are indicators of not only systemic inflammation but also different malignancies [23]. One study reported the NLR is an independent prognostic factor for relapse-free survival following surgical resection in CIN patients [24]. Furthermore, preoperative PLR levels could be a marker for predicting recurrence/residual disease of HSIL after LEEP [24]. We found that the NLR and PLR did not affect the outcome of ALA-PDT treatment for HSIL.

ALA-PDT combined with CO_2_ laser assistance is being explored to enhance its effectiveness and improve patient tolerance. The CO_2_ laser itself has therapeutic benefits. Previous clinical studies have shown improved lesion response when a CO_2_ laser is used before PDT compared to ALA-PDT or laser treatment alone [25,26]. However, this study revealed that there was no significant difference between the ALA-PDT+ CO_2_ laser group and the ALA-PDT group.

These results can inform ALA-PDT treatment for HSIL/CIN2 patients in the future. For patients who smoke or are in a smoking environment for a long time, it is necessary to quit smoking or consciously stay away from the smoking environment before ALA-PDT treatment and maintain this until the lesions have regressed. For patients with sleep disorders, they should be helped to find the cause, through symptomatic and etiological treatment, to improve their quality of sleep. These steps require the joint efforts of patients, families, and doctors. Having regular telephone or home follow-up appointments and the timely monitoring of the lifestyle behaviors of study participants will contribute to the rigor of future clinical studies.

The major deficiencies of this study were the small, single-center sample and short follow-up, which may have caused bias. Furthermore, analysis of the clinicopathological characteristics was not detailed enough, and hierarchical analysis may help evaluate the influences of characteristics more comprehensively.

## 5. Conclusions

Overall, we examined the relationship between 13 clinicopathological characteristics and the impact of ALA-PDT on HSIL/CIN2 patients. Our findings indicated that smoking and sleep disorders are independent risk factors for failure in lesion regression, while age, NLR, PLR, drinking, HPV vaccine, HPV infection type, TCT results, history of giving birth and abortion, and CO_2_ laser combination therapy have no effect on lesion regression. No factors affecting hrHPV clearance were found. We suggest that careful consideration is given when evaluating ALA-PDT for patients who smoke and those with sleep disorders. To make informed treatment decisions for HSIL patients, further trials with larger sample sizes and longer follow-up periods are necessary to confirm these findings and explore additional prognostic factors for ALA-PDT.

## Figures and Tables

**Table 1:** Analysis of factors influencing the effect of ALA-PDT on HSIL/CIN2 regression.

Characteristics	Sum (*n* = 69)	Success in Lesion Regression (*n* = 60)	Failure in Lesion Regression (*n* = 9)	χ2/t	*p*/*p*′/ Fisher
Age [x ± s, years]	30.55 ± 7.25	30.63 ± 7.61	30.00 ± 4.44	0.243	0.809
NLR [x ± s]	2.14 ± 0.85	2.13 ± 0.89	2.20 ± 0.53	−0.207	0.837
PLR [x ± s]	128.75 ± 40.18	127.34 ± 40.37	138.18 ± 39.90	−0.752	0.455
current smoking [*n*, (%)]					
not	52 (75.4%)	48 (92.3%)	4 (7.7%)	3.586	0.058
yes	17 (24.6%)	12 (70.6%)	5 (29.4%)		
drinking history [*n*, (%)]					
not	58 (84.1%)	52 (89.7%)	6 (10.3%)	1.082	0.298
yes	11 (15.9%)	8 (72.7%)	3 (27.3%)		
sleeping disorders [*n*, (%)]					
not	49 (71%)	47 (95.9%)	2 (4.1%)	9.400	0.002
yes	20 (29%)	13 (65.0%)	7 (35.0%)		
HPV vaccination [*n*, (%)]					
not	54 (78.3%)	45 (83.3%)	9 (16.7%)	1.593	0.207
yes	15 (21.7%)	15 (100.0%)	0 (0.0%)		
HPV16/18 infection [*n*, (%)]					
not	25 (36.2%)	22 (88.0%)	3 (12.0%)	0.000	1.000
yes	44 (63.8%)	38 (86.4%)	6 (13.6%)		
multiple types of HPV infection [*n*, (%)]					
not	48 (69.6%)	43 (89.6%)	5 (10.4%)	0.349	0.554
yes	21 (30.4%)	17 (81.0%)	4 (19.0%)		
Thinpred cytologic test [*n*, (%)]					
≤ASCUS	40 (58%)	36 (90.0%)	4 (10.0%)	0.270	0.603
≥LSIL	29 (42%)	24 (82.8%)	5 (17.2%)		
history of giving birth [*n*, (%)]					
not	50 (72.5%)	43 (86.0%)	7 (14.0%)	0.000	1.000
yes	19 (27.5%)	17 (89.5%)	2 (10.5%)		
abortion history [*n*, (%)]					
not	48 (69.6%)	42 (87.5%)	6 (12.5%)	0.000	1.000
yes	21 (30.4%)	18 (85.7%)	3 (14.3%)		
combined with CO2 laser [*n*, (%)]					
not	44 (63.8%)	40 (90.9%)	4 (9.1%)	0.849	0.357
yes	25 (36.2%)	20 (80.0%)	5 (20.0%)		

NLR: neutrophil–lymphocyte ratio; PLR: platelets–lymphocyte ratio. Current smoking: a regular active smoker or exposure to environmental tobacco smoke. Non-current smokers had quit or never smoked regularly. Drinking history: participants engaged in drinking (≥14 drinks/wk for women) for a 30-day consecutive period and ≥2 heavy drinking days (defined as ≥4 drinks/d for women) in the 90 days preceding or during ALA-PDT. Sleep disorders: according to the International Classification of Sleep Disorders-3 (ICSD-3), insomnia disorders, sleep-related breathing disorders, central disorders of hypersomnolence, circadian rhythm sleep–wake disorders, sleep-related movement disorders, parasomnias, and other sleep disorders were defined as sleep disorders. HPV vaccination means a patient had received three shots of HPV vaccine before ALA-PDT, no matter whether the bivalent, quadrivalent, or nine-valent vaccine.

**Table 2:** Analysis of factors influencing the effect of ALA-PDT on hrHPV clearance.

Characteristics	Sum (*n* = 69)	Success in hrHPV Clearance (*n* = 44)	Failure in hrHPV Clearance (*n* = 25)	χ^2^/t	*p*/*p*′/ Fisher
Age [x ± s, years]	30.55 ± 7.25	30.77 ± 7.52	30.16 ± 6.89	0.335	0.739
NLR [x ± s]	2.14 ± 0.85	2.14 ± 0.84	2.15 ± 0.88	−0.083	0.934
PLR [x ± s]	128.75 ± 40.18	124.96 ± 40.93	135.42 ± 38.75	−1.040	0.302
current smoking [*n*, (%)]					
not	52 (75.4%)	36 (69.2%)	16 (30.8%)	2.726	0.099
yes	17 (24.6%)	8 (47.1%)	9 (52.9%)		
drinking history [*n*, (%)]					
not	58 (84.1%)	39 (67.2%)	19 (32.8%)	1.074	0.300
yes	11 (15.9%)	5 (45.5%)	6 (54.5%)		
sleeping disorders [*n*, (%)]					
not	49 (71.0%)	34 (69.4%)	15 (30.6%)	2.311	0.128
yes	20 (29.0%)	10 (50.0%)	10 (50.0%)		
HPV vaccination [*n*, (%)]					
not	54 (78.3%)	34 (63.0%)	20 (37.0%)	0.070	0.792
yes	15 (21.7%)	10 (66.7%)	5 (33.3%)		
HPV16/18 infection [*n*, (%)]					
not	25 (36.2%)	19 (76%)	6 (24%)	2.539	0.111
yes	44 (63.8%)	25 (58.6%)	19 (43.2%)		
multiple types of HPV infection [*n*, (%)]					
not	48 (69.6%)	32 (66.7%)	16 (33.3%)	0.574	0.449
yes	21 (30.4%)	12 (57.1%)	9 (42.9%)		
Thinpred cytologic test [*n*, (%)]					
≤ASCUS	40 (58.0%)	25 (62.5%)	15 (37.5%)	0.066	0.797
≥LSIL	29 (42.0%)	19 (65.5%)	10 (34.5%)		
history of giving birth [*n*, (%)]					
not	50 (72.5%)	33 (66.0%)	17 (34.0%)	0.391	0.532
yes	19 (27.5%)	11 (57.9%)	8 (42.1%)		
abortion history [*n*, (%)]					
not	48 (69.6%)	32 (66.7%)	16 (33.3%)	0.574	0.449
yes	21 (30.4%)	12 (57.1%)	9 (42.9%)		
combined with CO_2_ laser [*n*, (%)]					
not	44 (63.8%)	28 (63.6%)	16 (36.4%)	0.001	0.976
yes	25 (36.2%)	16 (64.0%)	9 (36.0%)		

**Table 3:** Univariate and multivariate regression analysis of factors influencing the effect of ALA-PDT on HSIL/CIN2 regression.

	Univariate Analysis		Multivariate Analysis	
Characteristics	*p*	OR (95% CI)	*p*	OR (95% CI)
age	0.806	0.987 (0.891–1.093)		
NLR	0.834	1.089 (0.489–2.427)		
PLR	0.451	1.006 (0.990–1.023)		
current smoking	0.031	5.000 (1.162–21.509)	0.046	5352.87 (1.15–2.48 × 10^7^)
drinking history	0.142	3.250 (0.674–15.671)		
sleeping disorders	0.003	12.654 (2.341–68.387)	0.039	5466.59 (1.58–1.90 × 10^7^)
HPV vaccination	0.998	0.000 (0.000–0.000)		
HPV16/18 infection	0.846	1.158 (0.263–5.097)		
multiple types of HPV infection	0.334	2.024 (0.484–8.453)		
TCT	0.383	1.875 (0.457–7.700)		
history of giving birth	0.703	0.723 (0.136–3.834)		
abortion history	0.840	1.167 (0.262–5.186)		
combined with CO_2_ laser	0.206	2.500 (0.604–10.344)		

**Table 4:** Univariate regression analysis of factors influencing the effect of ALA-PDT on hrHPV clearance.

	Univariate Analysis	
Characteristics	*p*	OR (95% CI)
age	0.734	0.988 (0.922–1.059)
NLR	0.933	1.025 (0.575–1.827)
PLR	0.301	1.007 (0.994–1.019)
current smoking	0.104	2.531 (0.826–7.756)
drinking history	0.177	2.463 (0.666–9.105)
sleeping disorders	0.133	2.267 (0.780–6.585)
HPV vaccination	0.792	0.850 (0.254–2.843)
HPV16/18 infection included	0.116	2.407 (0.805–7.191)
multiple types of HPV infection	0.450	1.500 (0.524–4.296)
TCT	0.797	0.877 (0.323–2.380)
history of giving birth	0.532	1.412 (0.478–4.168)
abortion history	0.450	1.500 (0.524–4.296)
combined with CO_2_ laser	0.976	0.984 (0.354–2.735)

## Data Availability

The original contributions presented in the study are included in the article, further inquiries can be directed to the corresponding authors.
